# Synergistic and Offset Effects of Fungal Species Combinations on Plant Performance

**DOI:** 10.3389/fmicb.2021.713180

**Published:** 2021-09-13

**Authors:** Yoshie Hori, Hiroaki Fujita, Kei Hiruma, Kazuhiko Narisawa, Hirokazu Toju

**Affiliations:** ^1^Center for Ecological Research, Kyoto University, Kyoto, Japan; ^2^Graduate School of Arts and Sciences, Multi-Disciplinary Sciences Life Sciences, The University of Tokyo, Tokyo, Japan; ^3^College of Agriculture, Ibaraki University, Mito, Japan

**Keywords:** biodiversity, endophytes, microbial functions, species interactions, symbiosis, plant-associated microbiomes, plant–fungus interactions, microbe–microbe interactions

## Abstract

In natural and agricultural ecosystems, survival and growth of plants depend substantially on residing microbes in the endosphere and rhizosphere. Although numerous studies have reported the presence of plant-growth promoting bacteria and fungi in below-ground biomes, it remains a major challenge to understand how sets of microbial species positively or negatively affect plants’ performance. By conducting a series of single- and dual-inoculation experiments of 13 plant-associated fungi targeting a Brassicaceae plant species (*Brassica rapa* var. *perviridis*), we here systematically evaluated how microbial effects on plants depend on presence/absence of co-occurring microbes. The comparison of single- and dual-inoculation experiments showed that combinations of the fungal isolates with the highest plant-growth promoting effects in single inoculations did not have highly positive impacts on plant performance traits (e.g., shoot dry weight). In contrast, pairs of fungi with small/moderate contributions to plant growth in single-inoculation contexts showed the greatest effects on plants among the 78 fungal pairs examined. These results on the offset and synergistic effects of pairs of microbes suggest that inoculation experiments of single microbial species/isolates can result in the overestimation or underestimation of microbial functions in multi-species contexts. Because keeping single-microbe systems under outdoor conditions is impractical, designing sets of microbes that can maximize performance of crop plants is an important step for the use of microbial functions in sustainable agriculture.

## Introduction

Plants in natural and agricultural ecosystems are associated with diverse taxonomic groups of microbes, forming both positive and negative interactions with the microbiomes (Lundberg et al., 2012; Peay et al., 2016; Busby et al., 2017; Toju et al., 2018b). In particular, bacteria and fungi found within and around root systems have been reported as key determinants of plants’ survival and growth (Hiruma et al., 2016, 2018; Castrillo et al., 2017; Trivedi et al., 2020). A number of rhizosphere bacteria, for example, are known to stimulate plants’ growth by producing phytohormones (Lugtenberg and Kamilova, 2009; Bhattacharyya and Jha, 2012; Finkel et al., 2020). Mycorrhizal fungi are ancient symbionts of land plants (Remy et al., 1994; Taylor et al., 1995), providing soil phosphorus and/or nitrogen to their hosts (Richardson et al., 2009; Tedersoo et al., 2010; Jansa et al., 2019). Moreover, a growing number of studies have shown that diverse clades of endophytic and soil fungi support host plants by provisioning inorganic/organic forms of nutrients (Usuki and Narisawa, 2007; Newsham, 2011; Hiruma et al., 2016), activating plant immune systems (van Wees et al., 2008; Pieterse et al., 2014), and suppressing populations of pathogens/pests in the rhizosphere (Narisawa et al., 2004; Khastini et al., 2012; Gu et al., 2020). Thus, developing scientific bases for maximizing the benefits from those plant-associated microbiomes is an essential step for fostering sustainable crop production and restoring forest/grassland ecosystems (Bulgarelli et al., 2013; Carlström et al., 2019; Wagg et al., 2019; Saad et al., 2020).

One of the major challenges in utilizing plant-associated microbial functions is to design sets of microbial species/isolates (Vorholt et al., 2017; Paredes et al., 2018; Toju et al., 2018a; Wei et al., 2019). While a single microbial species or isolate can have specific functions in promoting plant growth, broader ranges of positive effects on plants are potentially obtained by introducing multiple microbial species/isolates (Wang et al., 2011; Ważny et al., 2018; He et al., 2020). For example, a fungal species degrading organic nitrogen (Newsham, 2011) and another fungus suppressing soil pathogens (Vinale et al., 2008) may collectively provide plants with a broader spectrum of physiological functions than each of them alone, potentially having additive or synergistic effects on the growth of their hosts. Meanwhile, sets of microbes trying to colonize the plant endosphere or rhizosphere may compete for resources/space (Kennedy et al., 2009; Werner and Kiers, 2015; Toju et al., 2016) or inhibit each other’s growth (Helfrich et al., 2018), making their impacts on host plants more negative than that observed under single-inoculation conditions (i.e., offset effects) (Nelson et al., 2018). Given that multiple microbial species inevitably interact with a single plant in agroecosystems (Toju et al., 2018a), knowledge of those synergistic and offset effects in plant-associated microbiomes is crucial for optimizing microbial functions in crop production.

A starting point for designing sets of microbes is to use the information of single-inoculation assays, in each of which a single microbial species/isolate is introduced to a target plant species/variety (Ahmad et al., 2008; Harbort et al., 2020). Through this initial assay, respective species/isolates are scored in terms of their functions (e.g., plant-growth promotion effects) under single-inoculation conditions (Nara, 2006; Dai et al., 2008; Taurian et al., 2010; Tsolakidou et al., 2019). The next step is to consider how these single-inoculation scores can be used for designing sets of microbes that potentially promote plant growth in synergistic ways. As the number of combinations inflates with that of constituent species/isolates [e.g., {N × (N – 1)}/2 combinations in two-species systems], prioritizing candidate species/isolate combinations based on single-inoculation results is an important step (Paredes et al., 2018; Toju et al., 2018a, 2020). The simplest way of exploring best sets of microbes is to combine microbes with highest single-inoculation scores. This strategy of combining microbes in highest ranks is promising if synergistic (or additive) effects are common in plant-associated microbiomes. In contrast, if offset effects of multiple microbes on plant performance are ubiquitous, alternative strategies for exploring species/isolate combinations are required to maximize benefits from plant-associated microbiomes. Thus, knowledge of the prevalence and intensity of such synergistic and offset effects is essential in synthetic microbiome studies. Nonetheless, although there have been studies reporting synergistic/offset effects of multiple plant-associated microbes (Han and Lee, 2006; Wang et al., 2011; Ważny et al., 2018; He et al., 2020), experimental studies systematically evaluating the commonness of those effects are scarce.

In this study, we tested the hypothesis that synergistic effects on plant growth are common in below-ground fungal biomes in a series of single- and dual-inoculation experiments. By using 13 plant-associated fungal species belonging to various taxonomic groups, we first evaluated their basic effects on plant growth in a single-inoculation experiments with a Brassicaceae species (*Brassica rapa* var. *perviridis*). We also performed dual-inoculation experiments for all the 78 possible combinations of the fungal species and then evaluated the performance of the combinations in light of single-inoculation results. The data then provided a platform for testing whether plant-growth promoting effects exceeding those of all the single-inoculation conditions are attainable under dual-inoculation conditions. Overall, this study provides a basis for understanding to what extent plant-growth promotion effects of microbiomes can be expected from the information of single-species inoculations, illuminating the potential importance of “non-additivity” in multi-microbe contexts.

## Materials and Methods

### Fungal Isolates for Inoculation Experiments

In the inoculation experiments detailed below, we used diverse fungal species isolated from plant roots (Table 1). Among the 13 fungal isolates used (Table 1 and Supplementary Data 1), some are reported as endophytic fungi promoting plant growth [e.g., *Colletotrichum tofieldiae*, *Cladophialophora chaetospira*, and *Veronaeopsis simplex*] in previous studies (Usuki and Narisawa, 2007; Hiruma et al., 2016; Guo et al., 2018). In addition, a species of *Trichoderma* with growth-promotion effects on tomato (*Solanum lycopersicum*) and *Brassica* plants (Toju et al., 2020) was used in the experiment. In addition, various taxonomic groups of fungal isolates were retrieved from the ca. 3,500 fungal isolates maintained in the culture collection of Centre for Ecological Research, Kyoto University (Supplementary Data 1). The 13 isolates examined in this study were selected so that not only well-characterized plant-growth-promoting fungi but also fungi with potentially negative or nearly neutral effects on plants were targeted in the experiments.

**TABLE 1:** Summary of fungal isolates used in the inoculation experiments.

**Isolate**	**Abbreviation**	**Phylum**	**Class**	**Order**	**Family**	**Genus**	**Guild**	**Blast top-hit**	**E value**	**Per. Ident**	**Accession**
*Phoma* sp. KUCER00000052	pho_0052	Ascomycota	Dothideomycetes	Pleosporales	Didymellaceae	*Phoma*	PES	*Phoma leveillei*	9.00E-123	99.6%	KY827373.1
*Alternaria* sp. KUCER00001239	alt_1239	Ascomycota	Dothideomycetes	Pleosporales	Periconiaceae	*Alternaria*	PES	*Alternaria broccoli-italicae*	2.00E-123	100.0%	MH374617.1
*Curvularia* sp. KUCER00000077	cur_0077	Ascomycota	Dothideomycetes	Pleosporales	Periconiaceae	*Curvularia*	P	*Curvularia coatesiae*	4.00E-126	100.0%	MK804384.1
*Setosphaeria* sp. KUCER00000031	set_0031	Ascomycota	Dothideomycetes	Pleosporales	Periconiaceae	*Setosphaeria*	PE	*Setosphaeria pedicellata*	1.00E-126	100.0%	LT837452.1
*Stemphylium* sp. KUCER00000804	ste_0804	Ascomycota	Dothideomycetes	Pleosporales	Periconiaceae	*Stemphylium*	PS	*Stemphylium lycopersici*	2.00E-125	100.0%	MN386223.1
*Veronaeopsis simplex* Y34	ver_0232	Ascomycota	Dothideomycetes	Venturiales	Sympoventuriaceae	*Veronaeopsis*	E	*Veronaeopsis simplex*	5.00E-125	100.0%	MH865233.1
*Cladophialophora chaetospira* M4006	cla_0230	Ascomycota	Eurotiomycetes	Chaetothyriales	Herpotrichiellaceae	*Cladophialophora*	E	*Cladophialophora chaetospira*	3.00E-123	99.6%	LC077702.1
*Aspergillus* sp. KUCER00000917	asp_0917	Ascomycota	Eurotiomycetes	Eurotiales	Aspergillaceae	*Aspergillus*	S	*Aspergillus terreus*	7.00E-124	99.6%	MH124236.1
*Colletotrichum tofieldiae* MAFF 712334	col_0223	Ascomycota	Sordariomycetes	Glomerellales	Glomerellaceae	*Colletotrichum*	PE	*Colletotrichum tofieldiae*	2.00E-125	100.0%	KX069824.1
*Trichoderma* sp. KUCER00000218	tri_0218	Ascomycota	Sordariomycetes	Hypocreales	Hypocreaceae	*Trichoderma*	PFES	*Trichoderma asperellum*	5.00E-125	100.0%	MT530021.1
*Fusarium* sp. KUCER00000983	fus_0983	Ascomycota	Sordariomycetes	Hypocreales	Nectriaceae	*Fusarium*	PES	*Fusarium oxysporum*	5.00E-125	100.0%	MT610995.1
*Tolypocladium* sp. KUCER00000289	tol_0289	Ascomycota	Sordariomycetes	Hypocreales	Ophiocordycipitaceae	*Tolypocladium*	FE	*Tolypocladium album*	9.00E-123	99.6%	LC386577.1
*Mucor* sp. KUCER00000113	muc_0113	Mucoromycota	–	Mucorales	Mucoraceae	*Mucor*	S	*Mucor abundans*	1.00E-125	100.0%	MK164195.1

*For each fungal isolate, taxonomy, functional guild information inferred by the FUNGuild database (P, plant pathogen; F, fungal pathogen; E, endophyte; S, saprophyte), and NCBI BLAST top-hit results of the ITS sequences are indicated for each isolate. See Supplementary Data 1 for detailed information of the isolates.*

In addition to observation under a microscope, the fungal isolates were identified based on the DNA sequencing of the internal transcribed spacer (ITS) region (DDBJ/ENA/NCBI accession number; LC632034-LC632046): all the ITS sequences matched NCBI database sequences of known fungal species with E-values less than 9.0 × 10^23^ (Table 1 and Supplementary Data 1). Putative functional groups of these fungi were inferred based on the taxonomic information using the FUNGuild program (Nguyen et al., 2016) as shown in Table 1. Note that such profiling information based on ecological guild databases should be interpreted with caution: even in a fungal genus embracing a number of plant pathogenic species, some species can have positive impacts on plants (Radhakrishnan et al., 2015; Hiruma et al., 2016).

### Fungal Inocula

Prior to the inoculation experiments, fungal inocula were prepared by modifying the protocols of some previous studies (Harsonowati et al., 2020; Toju et al., 2020) as detailed below. Each of the 1.3-L high-density polyethylene bags with air-conditioning filters (Shinkon Co. Ltd., Minokamo) was filled with the mixture of 60-cm^3^ wheat bran (Tamagoya Shoten), 60-cm^3^ rice bran, 180-cm^3^ leaf mold (Akagi Gardening Co., Ltd.), and 70-mL distilled water. The filled culture bags were sealed with a heat sealer (ANT-300, AS ONE Corporation, Osaka) and autoclaved three times at 121°C (103.7 kPa) for 30 min with 24 h intervals. For each fungal isolate, approximately ten pieces of mycelial disks (8.0 mm in diameter) were then transferred from 1/2 CMMY medium (cornmeal agar, 8.5 g/L; malt extract, 10.0 g/L; yeast extract, 1.0 g/L) (Becton, Dickinson and Co.) to the autoclaved substrate and the fungal culture bag was incubated at room temperature (approximately 25°C) for 10–21 days until it was filled with mycelia. In addition to the 13 fungal inocula, a mock inoculum without fungi was prepared as a control.

Each of the fungal/control inocula was mixed with autoclaved potting soil [one round of 121°C (103.7 kPa) for 20 min] consisting mainly of organic materials such as fermented bark, peat moss, and coconut peat [“Gin-no-tsuchi”; Total N, 4,100 mg/kg; P, 2,706 mg/kg; K, 2,823 mg/kg; pH, 6.70; electrical conductivity, 0.73 mS/cm; Kanea Inc., Takamatsu] by the proportion of 1:9. The mixed soil was then transferred into plastic cell trays: the size of each cell in the trays was 49 mm × 49 mm × 56.5 mm.

### Inoculation Experiments

The “Komatsuna Wase” variety of *B. rapa* var. *perviridis* (Atariya Noen Co. Ltd., Katori) was used as the target plant in the inoculation experiments because its fast-growing property was convenient for the assay. Before inoculation, the seeds of *Brassica* were surface sterilized by being shaken in 70% ethanol solution for 1 min and then in 1% sodium hypochlorite solution for 1.5 min. The seeds were then rinsed three times in distilled water. They were subsequently placed on 1% agar petri dishes and incubated at 23°C in the dark for 24–26 h until rooting. The rooted seeds were transferred to the inoculum-mixed soil on the following day: two seeds were introduced into each of the 20–35 replicate cells for each single inoculation experiment (Supplementary Data 2). The cell trays were maintained in the laboratory with the 16hL/8hD light condition at 25°C. The plants were watered 3–4 times a week. The locations of the cell trays were rotated to equalize plants’ growing conditions.

In addition to the above single-inoculation experiments, dual-inoculation experiments were performed for all the 78 possible combinations of the 13 fungal isolates. For each pair of fungal isolates, their inocula were mixed by the proportion of 1:1, collectively constituting 1/10 volume of the total soil volume within the cell pots. Two *Brassica* seeds were then introduced into each of the replicate cell pots and they were kept under laboratory conditions as detailed above. Due to the large number of treatments and replicates as well as the limited spatial capacity of the laboratory, the inoculation experiments were split into several experimental rounds (up to 13 single/dual/control treatments per round; see Supplementary Data 2 for the information of experimental rounds). To take into account potential difference of micro-environmental conditions among the experimental rounds, a control (mock inoculum) treatment was included in every round in order to standardize plant growth responses throughout the study (see below for the calculation of a standardized growth index).

After 7 days, the ratio of geminating seeds to introduced seeds (i.e., germination rate) was recorded for each single/dual/control treatment. The seedlings were randomly thinned to one seedling per cell and they were kept under the same environmental conditions for another 2 weeks.

### Plants’ Growth Responses

The 21-day old *Brassica* plant samples were harvested to evaluate their responses to fungal inoculations. For all the replicate samples, shoot dry weight (above-ground biomass) and the number of mature leaves (>20 mm in length) were recorded. For the measurement of shoot dry weight, plant samples were oven-dried at 60°C for at least 72 h. Leaves longer than 20 mm were also subjected to SPAD measurements to infer chlorophyll concentrations using a SPAD-502Plus meter (Konica Minolta, Inc., Tokyo) (Netto et al., 2005; Zhu et al., 2012). For each of the randomly-selected 15 plant samples per treatment, the SPAD readings at three points were averaged. While shoot dry weight and the number of mature leaves are metrics of plant total biomass, SPAD readings (chlorophyll concentrations) are often regarded as (weak) indicators of foliar nitrogen concentrations (Chang and Robison, 2003; Esfahani et al., 2008).

To standardize the variables representing plants’ responses to fungal inoculations, we calculated a standardized growth index as follows:


(1) $$\begin{array}{c} S\,G_{T}\,\left(i\right)=\frac{X_{T}\,\left(i\right)-\bar{X_{C}}}{S\,D_{C}}, \end{array}$$

where *X*_*T*_(*i*) is a measurement of a target trait of a plant sample *i* in a target single-/dual-inoculation treatment, while $\bar{X_{C}}$ and *SD*_*C*_ are the mean and standard deviation of plant traits (variables) observed in the control samples of the focal experimental round, respectively. In terms of basic statistics assuming the Gaussian distribution, the standardized growth index [*SG*_*T*_(*i*)] values less than −1.96 and those larger than 1.96 roughly represented plant performance outside the 95% confidence intervals of the control samples in the same experimental rounds, providing an intuitive criterion for comparing results within/across inoculation experiments (see Supplementary Figure 1 for relationship between the standardized growth index values and false discovery rates). This standardized growth index was calculated for each of the three plant variables representing plant performance: the number of mature leaves, shoot dry weight, and chlorophyll concentrations.

### Synergistic and Offset Effects

**FIGURE 1:**
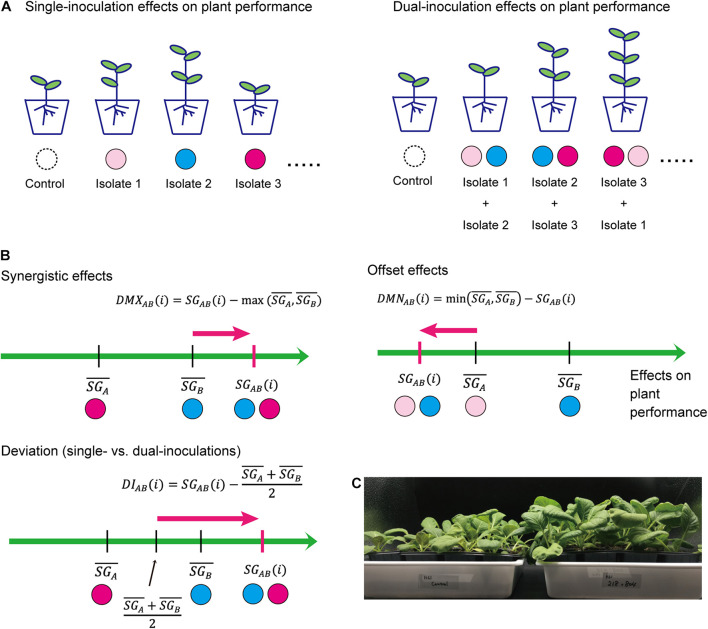
Evaluation of effects on plant performance. **(A)** Schema of single- and dual-inoculation assays. **(B)** Indexes for comparing single vs. dual inoculation effects. Along the axis of standardized growth index defined by the Equation (1), index values representing synergistic/offset effects on plants are calculated for each replicate plant sample for each pair of microbial (fungal) isolates [*DMX*_*AB*_(*i*) and *DMN*_*AB*_(*i*)]. Likewise, index values representing deviation of dual-inoculation effects from single-inoculation effects are obtained [*DI*_*AB*_(*i*)]. **(C)** Example of inoculation experiments. *Brassica* plants inoculated with two fungal isolates (tri_0218 × ste_0804; right) and those without fungal inoculations (control; left).

Based on the standardized growth index, we evaluated potential synergistic effects in dual inoculations of two fungal isolates in comparison to single-inoculation results (Figure 1). For a replicate plant sample inoculated with a pair of fungal isolates A and B, the index representing deviation from the maximum effects in single inoculations is calculated as follows:


(2) $$\begin{array}{c} D\,M\,X_{A\,B}\,\left(i\right)=S\,G_{A\,B}\,\left(i\right)-\max\left(\bar{S\,G_{A}},\bar{S\,G_{B}}\right), \end{array}$$

where *SG*_*AB*_(*i*)is the standardized growth index of a replicate plant in the dual inoculation treatment, while $\bar{S\,G_{A}}$ and $\bar{S\,G_{B}}$ are means of standardized growth index values for the single inoculation of fungal isolates A and B, respectively. By definition, when there are synergistic effects of the presence of two fungal isolates [i.e., $\bar{S\,G_{A\,B}}>\text{max}\,\left(\bar{S\,G_{A}},\bar{S\,G_{B}}\right)$], the mean of the deviation index over replicate plant samples$(\bar{D\,M\,X_{A\,B}}$) is larger than zero. Likewise, to evaluate offset effects in dual inoculations of two fungal isolates, an index representing deviation from the minimum effects in single inoculations was defined as follows:


(3) $$\begin{array}{c} D\,M\,N_{A\,B}\,\left(i\right)=\min\left(\bar{S\,G_{A}},\bar{S\,G_{B}}\right)-S\,G_{A\,B}\,\left(i\right). \end{array}$$

When there are offset effects [i.e.,$\bar{S\,G_{A\,B}}<\text{min}\,\left(\bar{S\,G_{A}},\bar{S\,G_{B}}\right)$] for a focal pair of fungi, mean of the offset effect index over replicate samples ($\bar{D\,M\,N_{A\,B}}$) is larger than zero.

We further developed a simple index for evaluating deviations of observed dual-inoculation results from those expected as intermediate results of single inoculations. For a pair of fungal isolates A and B, the index for deviation from intermediate effects is calculated as follows:


(4) $$\begin{array}{c} D\,I_{A\,B}\,\left(i\right)=S\,G_{A\,B}\,\left(i\right)-\frac{\bar{S\,G_{A}}+\bar{S\,G_{B}}}{2}. \end{array}$$

If the plant-growth promoting effects under the presence of two fungal isolates is close to what is expected as the intermediate results of the single inoculation assays of the two isolates, the index for deviation from intermediate effects [*DI*_*AB*_(*i*)] or its mean over replicate samples ($\bar{D\,I_{A\,B}}$) is likely to have a value around zero.

### Nonlinearity of Fungus–Fungus Combinations

For each pair of fungal isolates (A and B), an analysis of variance (ANOVA) model of standardized growth index was constructed by including the presence/absence of isolate A, the presence/absence of isolate B, and the interaction term of the two (i.e., isolate A × isolate B) as explanatory variables. Then, across the 78 fungal pairs examined, *F* values of the isolate A × isolate B term were compared as indicators of how combinations of the two fungal isolates had “nonlinear” effects on plant performance traits. We then examined how the nonlinearity measures of fungal pairs are associated with the abovementioned index values representing deviations of observed dual-inoculation results from those expected as intermediate results of single inoculations ($\bar{D\,I_{A\,B}}$).

All the calculations of the above indexes and statistical analyses were performed using the R base ver. 3.6.0 (R Core Team, 2020).

## Results

### Germination Rates

The gemination rates of *Brassica* plants varied within single inoculation treatments and within dual inoculation treatments (Supplementary Figure 2). Meanwhile, the rates were generally higher in dual inoculation treatments than in single inoculation treatments (Welch’s test; *t* = −3.97, df = 13.6, *P* = 0.0015).

### Plants’ Growth Responses

**FIGURE 2:**
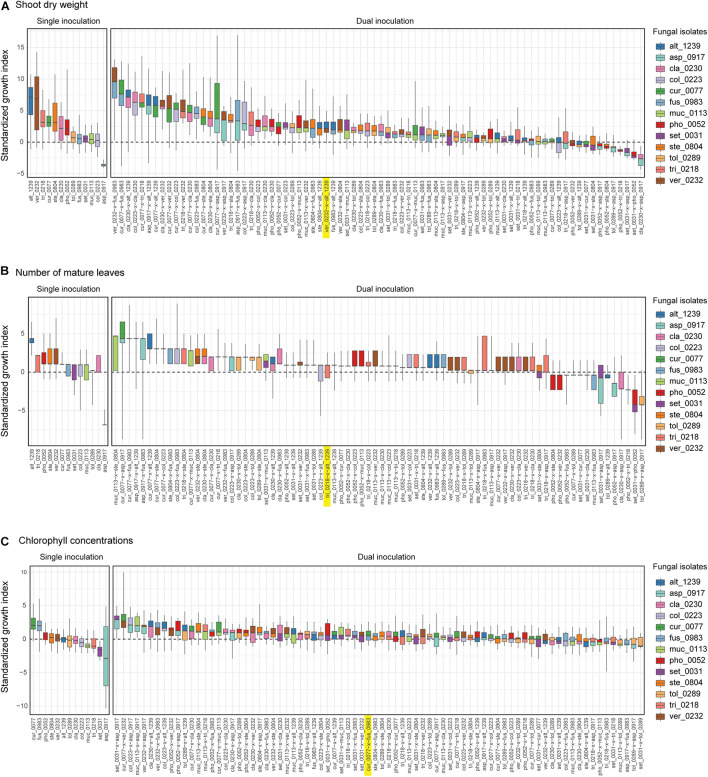
Single- and dual-inoculation effects on *Brassica* plants. **(A)** Standardized growth index in terms of shoot dry weight. For respective single- and dual-inoculation experiments, 25% quantiles, medians, and 75% quantiles are displayed as boxes and the ranges from the maximum to minimum values are shown as bars. See Table 1 for the abbreviation of fungal isolates. The combination of the fungal species with the largest positive effects on *Brassica* plants in single inoculation experiments is highlighted. **(B)** Standardized growth index in terms of the number of mature leaves. **(C)** Standardized growth index in terms of chlorophyll concentrations.

For all the three plant performance variables (shoot dry weight, the number of mature leaves, and SPAD), the single inoculation effects on *Brassica* plants differed significantly among the 13 fungal isolates examined (Table 2). For example, the mean standardized growth index for *V. simplex* Y34 and *Alternaria* sp. KYOCER00001239 were, on average, ca. seven-fold larger than the standard deviation of control sample’s growth (i.e., $\bar{S\,G_{T}}$ *>* 7) in terms of shoot dry weight, indicating high growth-promoting effects of those fungi on *Brassica* plants (Figure 2A). In addition, *C. chaetospira* M4006, *Trichoderma* sp. KYOCER00000218, *Curvularia* sp. KYOCER00000077, *Phoma* sp. KYOCER00000052, and *Stemphylium* sp. KYOCER00000804 showed high plant growth promoting effects (Figure 2A). In contrast, *C. tofieldiae* MAFF 712334, *Mucor* sp. KYOCER00000113, *Setosphaeria* sp. KYOCER00000031, *Fusarium* sp. KYOCER00000983, and *Tolypocladium* sp. KYOCER00000289 displayed weak or almost neutral effects on plant growth and *Aspergillus* sp. KYOCER00000917 had negative impacts on the *Brassica* plants (Figure 2A). When the number of mature leaves was used as a metric of plant performance, *Alternaria* sp. KYOCER00001239 and *Aspergillus* sp. KYOCER00000917 turned out to have strongly positive and negative effects, respectively (Figure 2B). Meanwhile, the effects of other fungal isolates were moderately positive or neutral (Figure 2B).

**TABLE 2:** Analysis of variance (ANOVA) results of single- and dual-inoculation experiments.

ANOVA model	df	*F*	*P*
**Single inoculation (across 13 fungal isolates)**			
Shoot dry weight	12	41.6	<0.0001
Number of mature leaves	12	127.7	<0.0001
Chlorophyll concentrations	12	12.0	<0.0001
**Dual inoculation (across 78 fungal pairs)**			
Shoot dry weight	77	25.7	<0.0001
Number of mature leaves	77	23.1	<0.0001
Chlorophyll concentrations	77	5.7	<0.0001

*For each of the three plant performance variables, an ANOVA model was constructed to examine the variation across single- or dual-inoculation treatments.*

In the dual inoculation experiments, the pair of the fungal isolates that exhibited the greatest effects in single inoculation treatments (i.e., *V. simplex* Y34 and *Alternaria* sp. KYOCER00001239) had relatively weak positive effects on *Brassica* growth in terms of shoot dry weight (Figure 2A). Instead, the highest plant-growth promoting effects were observed for the combination of *V. simplex* Y34 and *Fusarium* sp. KYOCER00000983, which had neutral effects on plants in the single inoculation (Figure 2A). Highly positive effects on plants (e.g., $\bar{S\,G_{T}}$ > 5) were observed, as well, in *Curvularia–Fusarium*, *Cladophialophora*–*Alternaria*, *Colletotrichum*–*Cladophialophora*, *Aspergillus*–*Alternaria*, and *Cladophialophora*–*Veronaeopsis* pairs and several other pairs including *Curvularia* sp. KYOCER00000077: for these pairs, at least one partner had neutral to weakly positive performance in single inoculation treatments (Figure 2A).

**FIGURE 3:**
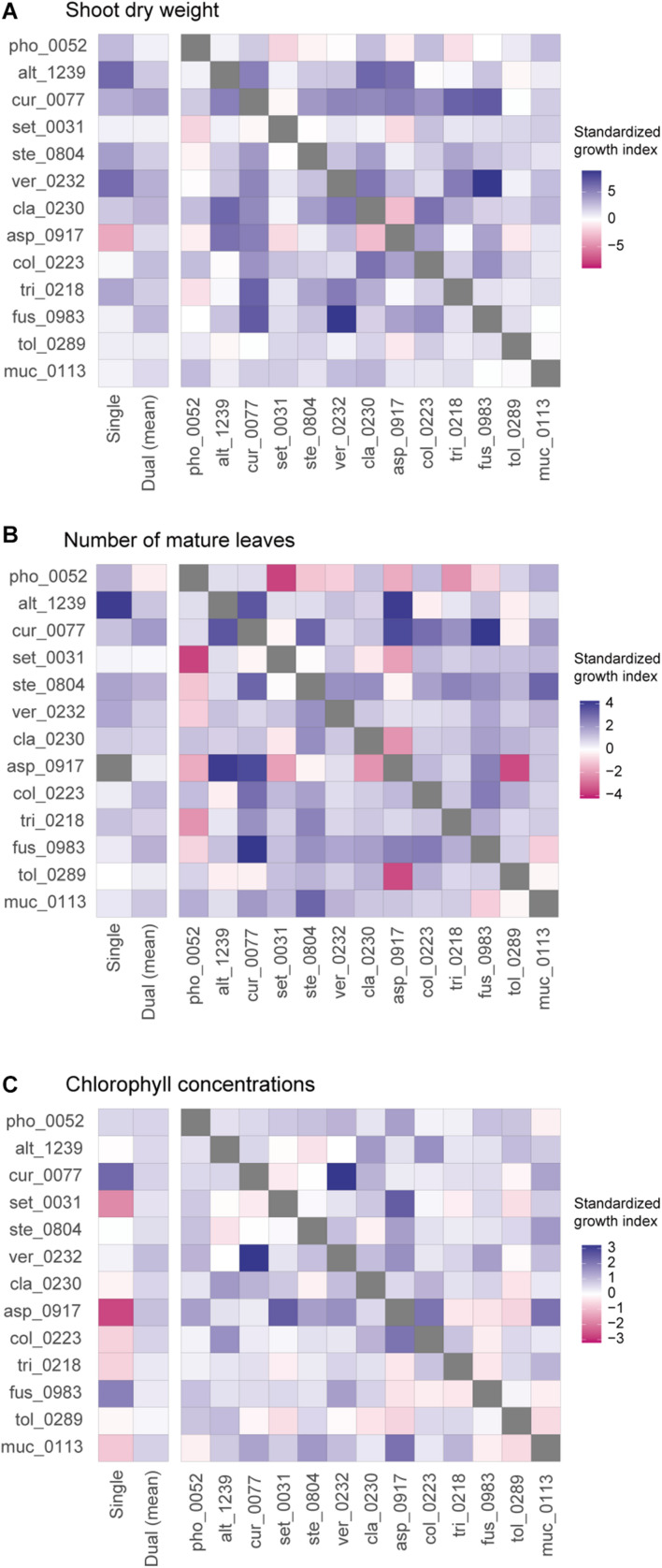
Pairwise representation of dual inoculation results. **(A)** Standardized growth index in terms of shoot dry weight for each pair of fungal isolates. Single-inoculation effects and mean effects across the dual inoculation assays are shown for each fungal isolate in the left. **(B)** Standardized growth index in terms of the number of mature leaves. **(C)** Standardized growth index in terms of chlorophyll concentrations.

In contrast to those combinations with relatively high plant-growth promoting effects (in the metrics of shoot dry weight and the number of mature leaves), *Aspergillus* sp. KYOCER00000917, which restricted plant growth in the single inoculation condition (Figures 2A,B), had negative impacts on plants in some of the 12 combinations with other fungal isolates (Figures 3A,B). However, their negative effects diminished in dual inoculations with some fungi such as *Alternaria* sp. KYOCER00001239 and *Curvularia* sp. KYOCER00000077 (Figures 3A,B). Results also showed that *Phoma* sp. KYOCER00000052, whose impacts on plants were positive in the single inoculation setting, inhibited plant growth in the presence of other fungi (Figures 3A,B).

When SAPD readings were used as metrics of plant performance, the *Curvularia* sp. KYOCER00000077 and *Fusarium* sp. KYOCER00000983 had relatively high positive effects on *Brassica* plants ($\bar{S\,G_{T}}$≈ 2), while *Setosphaeria* sp. KYOCER00000031 and *Aspergillus* sp. KYOCER00000917 had negative impacts (Figure 2C). Note that chlorophyll concentrations were weakly correlated with shoot dry weight and the number of mature leaves (Supplementary Figure 3). In the dual inoculation experiments, some fungal pairs including *Aspergillus* sp. KYOCER00000917 had relatively high positive effects on *Brassica* plants (Figure 3C) despite negative impacts of the *Aspergillus* isolate in a single-inoculation condition (Figure 2C). The pair of *Curvularia* and *Veronaeopsis* moderately increased chlorophyll concentrations as well (Figure 3C). Meanwhile, chlorophyll concentrations did not differ greatly from the control for most fungal pairs (Figure 2C).

For all the three plant performance variables examined, standardized growth index values of single inoculation experiments were uncorrelated with those averaged across dual inoculations for respective fungi (shoot dry weight, *r* = −0.09, *P* = 0.78; number of mature leaves *r* = 0.11, *P* = 0.71; SPAD, *r* = −0.41, *P* = 0.17; Figure 3). In other words, fungi with more positive effects on plants in single-inoculation experiments did not increased plant performance more efficiently. The experimental results also indicated that some combinations of fungi exhibited higher impacts on *Brassica* performance than that observed in all the single-inoculation settings (Figures 2A–C).

### Synergistic and Offset Effects

**FIGURE 4:**
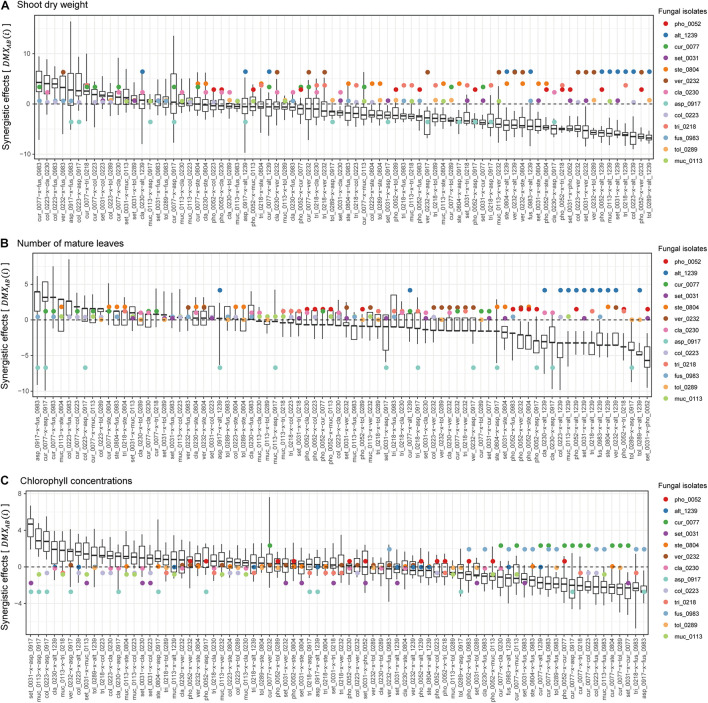
Synergistic effects observed in dual-inoculation experiments. **(A)** Synergistic effect index in terms of shoot dry weight. The index representing deviation of dual-inoculation effects from the maximum effects in single inoculations are shown for each pair of fungal isolates. Circles represent single-inoculation effects of respective fungal isolates. **(B)** Synergistic effect index in terms of the number of mature leaves. **(C)** Synergistic effect index in terms of chlorophyll concentrations.

Among the 78 combinations of fungal isolates, strong synergistic effects [$\bar{S\,G_{A\,B}}>\text{max}\,\left(\bar{S\,G_{A}},\bar{S\,G_{B}}\right)$] were observed in some pairs of fungi in terms of shoot dry weight (Figure 4A). The fungal combinations with the largest synergistic effects ($\bar{D\,M\,X_{A\,B}}$) consisted of *Curvularia* sp. KYOCER00000077 and *Fusarium* sp. KYOCER00000983, each of which had weakly or moderately positive impacts on plant growth in single inoculations. Large synergistic effects were detected in other pairs of fungi including fungi with moderate or weakly positive effects on plants (e.g., *Colletotrichum–Cladophialophora*, *Colletotrichum–Fusarium*, and *Veronaeopsis–Fusarium* pairs; Figure 4A). Similarly, for the number of mature leaves, fungal pairs with large synergistic effects involved fungi with weakly positive or even negative effects in single inoculations (Figure 4B). In terms of chlorophyll concentrations, pairs of fungi with negative impacts on plants under single-inoculation conditions had large synergistic effects (Figure 4C).

**FIGURE 5:**
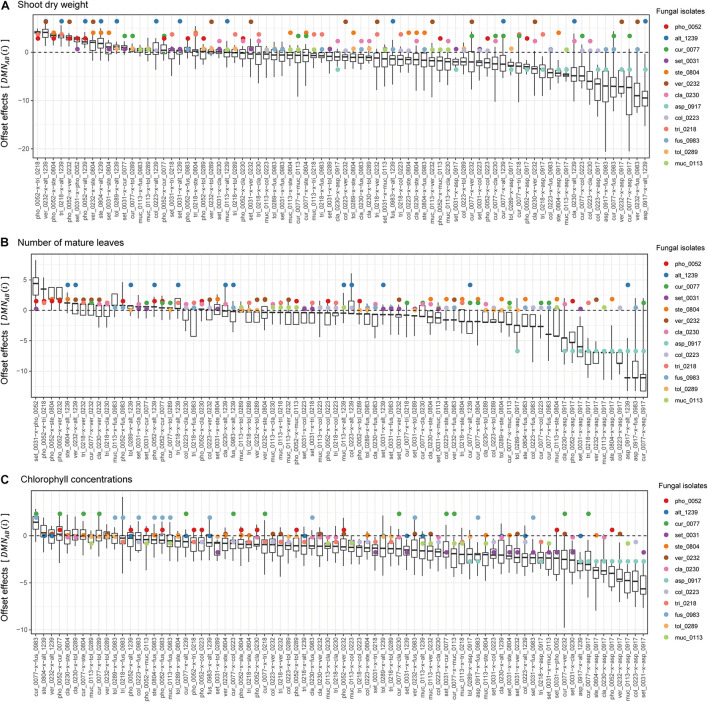
Offset effects observed in dual-inoculation experiments. **(A)** Offset effect index in terms of shoot dry weight. The index representing deviation of dual-inoculation effects from the minimum effects in single inoculations are shown for each pair of fungal isolates. Circles represent single-inoculation effects of respective fungal isolates. **(B)** Offset effect index in terms of the number of mature leaves. **(C)** Offset effect index in terms of chlorophyll concentrations.

In contrast to synergistic effects, offset effects [$\bar{S\,G_{A\,B}}<\text{min}\,\left(\bar{S\,G_{A}},\bar{S\,G_{B}}\right)$] were evident especially in the fungal pairs including fungi that had highly positive impacts on plant performance traits under single-inoculation conditions (Figure 5). In particular, the pairs of fungi with the largest positive effects (i.e., the *Veronaeopsis–Alternaria* pair) showed large offset effects (Figure 5).

**FIGURE 6:**
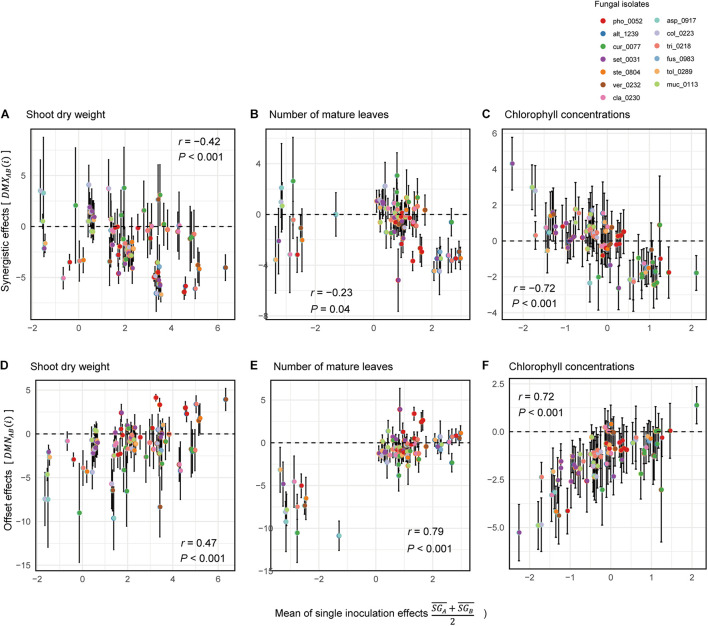
Relationship between single-inoculation effects and synergistic/offset effects. **(A)** Trends in synergistic effects in terms of shoot dry weight. For each pair of fungi, mean values of single inoculation effects of the target fungi (i.e., $\frac{\bar{S\,G_{A}}+\bar{S\,G_{B}}}{2}$) and index values of synergistic effects [i.e., *DMX*_*AB*_(*i*)] are shown at the horizontal and vertical axes, respectively. Error bars represent standard deviations of synergistic effects. **(B)** Trends in synergistic effects in terms of the number of mature leaves. **(C)** Trends in synergistic effects in terms of chlorophyll concentrations. **(D)** Trends in synergistic effects in terms of shoot dry weight. For each pair of fungi, mean values of single inoculation effects of the target fungi (i.e., $\frac{\bar{S\,G_{A}}+\bar{S\,G_{B}}}{2}$) and index values of offset effects [i.e., *DMN*_*AB*_(*i*)] are shown at the horizontal and vertical axes, respectively. **(E)** Trends in synergistic effects in terms of the number of mature leaves. **(F)** Trends in synergistic effects in terms of chlorophyll concentrations.

Across the 78 combinations of fungi, synergistic effects (i.e., $\bar{D\,M\,X_{A\,B}}$) decreased with increasing mean values of single inoculation effects of the target fungi (i.e., $\frac{\bar{S\,G_{A}}+\bar{S\,G_{B}}}{2}$) (Figures 6A–C). In other words, pairs of fungi that showed greater plant-performance increasing effects tended to have weaker synergistic effects. As expected by the trend in synergistic effects, offset effects were increased with increasing mean values of single inoculation effects of the target fungi (Figures 6D–F).

### Nonlinearity of Fungus–Fungus Combinations

**FIGURE 7:**
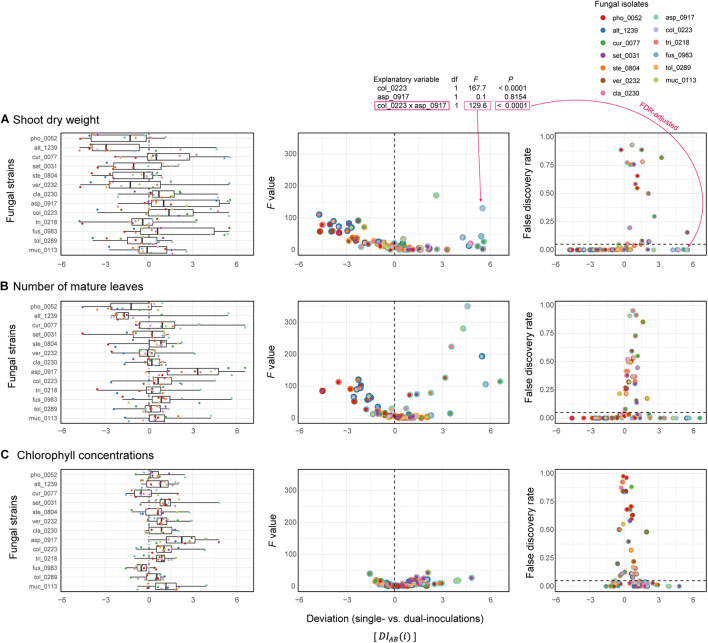
Deviations of observed dual-inoculation results from those expected as intermediate results of single inoculations. **(A)** Deviation index for shoot dry weight. The index values representing deviations of dual-inoculation effects from intermediate effects in single inoculations ($\bar{D\,I_{A\,B}\,\left(i\right)}$) are shown for each fungal isolate included in the target fungal pairs (left). For each fungal pair, *F* values of the isolate A × isolate B term in the ANOVA model (middle) and false discovery rate (FDR) values of the interaction term (right) are shown across the axis of the deviation index: FDR are calculated across the 78 fungal combinations examined. **(B)** Deviation index for the number of mature leaves. **(C)** Deviation index for chlorophyll concentrations.

Deviations of observed dual-inoculation results from those expected as intermediate results of single inoculations ($\bar{D\,I_{A\,B}}$) varied among fungal pairs (Figure 6). Higher absolute $\bar{D\,I_{A\,B}}$ values were indicative of nonlinearity in effects on plants for the particular fungus–fungus combinations as evaluated by a series of ANOVA models (Figure 7 and Supplementary Data 3).

## Discussion

By using taxonomically diverse plant-associated fungi, we here evaluated plant-growth promoting effects of pairs of fungal isolates in light of those observed in single-isolate inoculation experiments. The 13 fungal isolates differed greatly in their independent effects on *Brassica* plants (Figures 2, 3), providing an ideal opportunity for examining how the ranking of plant-growth promoting effects in single-inoculation contexts were related to that in multi-species (dual-inoculation) contexts (Figures 4–6). Such information of synergistic and offset effects in the presence of multiple microbial species is indispensable for understanding to what extent we can predict functions of microbial communities (microbiomes) from the datasets of single-species/isolate screening.

A series of single- and dual-inoculation experiments indicated that greater performance of plants were potentially obtained in multi-species than in single-species contexts (Figure 2). This result, itself, is consistent with previous reports of enhanced plant growth by specific pairs of bacteria/fungi (Han and Lee, 2006; Wang et al., 2011; Ważny et al., 2018; He et al., 2020). Meanwhile, our experiments on 78 combinations of fungi systematically suggested that pairs of microbes, each of which had greatly positive impacts on plant growth in single inoculations, could show minor effects on plants under multi-species conditions. For example, the strategy of combining the two species/isolates with highest performance in the single inoculation experiments (i.e., *V. simplex* and *Alternaria* sp. KYOCER00001239) did not result in high plant-growth promoting effects (Figure 3): rather, offset effects were observed in those “top × top” pairs (Figures 4–6). Thus, biological functions at the community (microbiome) level may be rarely maximized by the “bottom-up” exploration of sets of microbes based solely on single-inoculation experiments.

Our experiments also suggested that pairs of microbes with subordinate performance in single inoculation assays could show largest growth-promoting effects on plants (Figure 2). This result suggests that single-species/isolate screening does not always provide sufficient information for predicting microbial performance at the multi-species level (Toju et al., 2018a). Interestingly, the fungal pairs with highest synergistic effects in our experiment involved fungi in the genera *Fusarium* and *Curvularia* (Figure 4A), which were often described as plant pathogenic taxa (Michielse and Rep, 2009; Ma et al., 2013; Manamgoda et al., 2015). Basically, physiological effects on plants vary remarkably among species/isolates within taxa as evidenced by the presence of *Fusarium* and *Curvularia* species enhancing plant health and growth (Olivain et al., 2006; Nahalkova et al., 2008; Priyadharsini and Muthukumar, 2017). In fact, the *Fusarium* and *Curvularia* isolates examined in our study had positive effects on *Brassica* plants even in the single-inoculation assays (Figure 2). Moreover, the results of the dual inoculation experiments suggested that some fungi in these predominantly plant-pathogenic genera can have even greater positive effects on plants in combination with specific other fungi (Figures 2, 3). Our results on synergistic effects in multi-species contexts further illuminate the potential use of diverse endosphere/rhizosphere microbes whose biological functions have been underestimated in conventional screening of single inoculations.

The fact that microbial functions critically depend on combinations of microbial species/isolates highlight the importance of “bird’s-eye” views of designing microbiomes. Given that microbial functions at the community (multi-species) levels are not the simple sums/averages of functions in single-species contexts (Figures 2, 7), research strategies taking into account not only each microbe’s roles but also the nature of microbe–microbe interactions will provide platforms for optimization of microbiome functions (Agler et al., 2016; Toju et al., 2016; Banerjee et al., 2018). In this respect, interdisciplinary approaches integrating the observational, genomic, and metagenomic information of microbial functions (Bulgarelli et al., 2015; Levy et al., 2018; Ichihashi et al., 2020) with community ecological analyses of species interaction networks (Agler et al., 2016; van der Heijden and Hartmann, 2016; Toju et al., 2017) will help us explore highly functional and stable microbial sets among numerous candidate combinations of species (Paredes et al., 2018; Saad et al., 2020; Toju et al., 2020). In other words, information of microbial functions in single-species contexts is utilized by being combined with insights into dynamics and processes within microbiomes.

While the experiments conducted in this study provided a unique opportunity for systematically evaluating synergistic/offset effects of microbes on plants, the obtained datasets should be interpreted with caution given the following limitations. First, physiological mechanisms by which the examined fungi affected plant growth were unexplored in the current study. Although detailed physiological and/or molecular biological investigations have been done for some of the fungal species used in this study [e.g., *C. tofieldiae* (Hiruma et al., 2016), *V. simplex* (Guo et al., 2018), and *C. chaetospira* (Harsonowati et al., 2020)], metabolites and genes involved in the plant–fungus interactions are unknown for the remaining species. For more mechanistic understanding of interactions involving plants and multiple microbial species, we need to perform transcriptomic analyses targeting plants’ responses to each microbe as well as those comparing plants’ gene expression patterns between single- and multiple-symbiont conditions. Comparative transcriptomic analyses across experiments with different environmental conditions (e.g., soil nutrient concentrations) will provide essential insights into microbial functions as well. Second, the inoculation test based on single plant species precluded us from understanding how general synergistic/offset effects existed in plant–fungal biome interactions. Although some of the fungal taxa used in this study have been reported to interact with multiple families of plants (Hermosa et al., 2012; Toju et al., 2018b), impacts of endophytic/soil fungi on plants can vary depending on plant taxa and environmental conditions (Kiers et al., 2011; Pineda et al., 2013; Rudgers et al., 2020). Therefore, to gain more robust insights into synergistic/offset effects in interactions of plants and multiple microbial species/isolates, the reproducibility of the patterns observed in this study should be examined in inoculation experiments targeting diverse other plant species. Third, it is important to acknowledge that the complexity of the microbial sets examined in this study is minimal (i.e., two fungal species): different types of phenomena may be observed in combinations of three or more bacterial/fungal species (Durán et al., 2018; Paredes et al., 2018; Carlström et al., 2019; Wei et al., 2019). Moreover, it remains to be examined how we can increase microbial functions (e.g., host plant growth rates) by increasing the number of microbial species/isolates. The presence of microbial pairs outperforming single-microbe systems (Figure 2) leads to the working hypothesis that compatible sets of three or more microbial species yield greater functions than simpler communities by playing complementary roles. Meanwhile, it is expected that benefits of microbiomes do not increase linearly with increasing number of microbial species (i.e., saturating curves of benefits against increasing number of microbes) (van der Heijden et al., 1998), at least in terms of specific functions such as provisioning of soil phosphorus or blocking of soil pathogens.

We here showed that screening based on inoculations of single microbial species/isolates can result in the underestimation of the microbes that potentially have large plant-growth promoting effects in combinations with specific other microbes. Given that plants are inevitably associated with hundreds or more of microbial species in agricultural and natural ecosystems (Lundberg et al., 2012; Schlaeppi and Bulgarelli, 2015; van der Heijden and Hartmann, 2016; Thoms et al., 2021), such nonlinearity found in microbe–microbe associations deserve future intensive research. It may be important, for instance, to examine how antagonistic relationships between salicylic-acid- and jasmonic-acid-related plant physiological responses (Niki et al., 1998), which are activated by different types of bacteria/fungi (Robert-Seilaniantz et al., 2011) [but see Betsuyaku et al. (2018)], can result in such nonlinear effects of multiple microbes on plant performance. Interdisciplinary studies on relationships between microbiome compositions and their ecosystem-level functions are awaited toward the maximization of microbial functions for sustainable agriculture and ecosystem restoration.

## Data Availability Statement

The datasets presented in this study can be found in online repositories. The names of the repository/repositories and accession number(s) can be found below: DDBJ [accession: LC632034-LC632046]. Accession numbers for additional data relevant to this study can be found in the manuscript or Supplementary Material.

## Author Contributions

YH and HT designed the work. YH carried out the experiments. YH, HF, and HT analyzed the data. YH and HT wrote the manuscript based on discussion with HF, KH, and KN. All authors contributed to the article and approved the submitted version.

## Conflict of Interest

The authors declare that the research was conducted in the absence of any commercial or financial relationships that could be construed as a potential conflict of interest.

## Publisher’s Note

All claims expressed in this article are solely those of the authors and do not necessarily represent those of their affiliated organizations, or those of the publisher, the editors and the reviewers. Any product that may be evaluated in this article, or claim that may be made by its manufacturer, is not guaranteed or endorsed by the publisher.

## References

[B1] Agler M. T. Ruhe J. Kroll S. Morhenn C. Kim S. T. Weigel D. (2016). Microbial hub taxa link host and abiotic factors to plant microbiome variation. *PLoS Biol.* 14:e1002352. 10.1371/journal.pbio.1002352 26788878 PMC4720289

[B2] Ahmad F. Ahmad I. Khan M. S. (2008). Screening of free-living rhizospheric bacteria for their multiple plant growth promoting activities. *Microbiol. Res.* 163 173–181. 10.1016/j.micres.2006.04.001 16735107

[B3] Banerjee S. Schlaeppi K. van der Heijden M. G. A. (2018). Keystone taxa as drivers of microbiome structure and functioning. *Nat. Rev. Microbiol.* 16 567–576. 10.1038/s41579-018-0024-1 29789680

[B4] Betsuyaku S. Katou S. Takebayashi Y. Sakakibara H. Nomura N. Fukuda H. (2018). Salicylic acid and jasmonic acid pathways are activated in spatially different domains around the infection site during effector-triggered immunity in *Arabidopsis thaliana*. *Plant Cell Physiol.* 59 8–16. 10.1093/pcp/pcx181 29177423 PMC6012717

[B5] Bhattacharyya P. N. Jha D. K. (2012). Plant growth-promoting rhizobacteria (PGPR): emergence in agriculture. *World J. Microbiol. Biotechnol.* 28 1327–1350. 10.1007/s11274-011-0979-9 22805914

[B6] Bulgarelli D. Garrido-Oter R. Münch P. C. Weiman A. Dröge J. Pan Y. (2015). Structure and function of the bacterial root microbiota in wild and domesticated barley. *Cell Host Microbe* 17 392–403. 10.1016/j.chom.2015.01.011 25732064 PMC4362959

[B7] Bulgarelli D. Schlaeppi K. Spaepen S. van Themaat E. V. L. Schulze-Lefert P. (2013). Structure and functions of the bacterial microbiota of plants. *Ann. Rev. Plant Biol.* 64 807–838. 10.1146/annurev-arplant-050312-120106 23373698

[B8] Busby P. E. Soman C. Wagner M. R. Friesen M. L. Kremer J. Bennett A. (2017). Research priorities for harnessing plant microbiomes in sustainable agriculture. *PLoS Biol.* 15:e2001793. 10.1371/journal.pbio.2001793 28350798 PMC5370116

[B9] Carlström C. I. Field C. M. Bortfeld-Miller M. Müller B. Sunagawa S. Vorholt J. A. (2019). Synthetic microbiota reveal priority effects and keystone strains in the *Arabidopsis phyllosphere*. *Nat. Ecol. Evol.* 3 1445–1454. 10.1038/s41559-019-0994-z 31558832 PMC6774761

[B10] Castrillo G. Teixeira P. J. Paredes S. H. Law T. F. de Lorenzo L. Feltcher M. E. (2017). Root microbiota drive direct integration of phosphate stress and immunity. *Nature* 543 513–518. 10.1038/nature21417 28297714 PMC5364063

[B11] Chang S. X. Robison D. J. (2003). Nondestructive and rapid estimation of hardwood foliar nitrogen status using the SPAD-502 chlorophyll meter. *Forest Ecol. Manag.* 181 331–338. 10.1016/S0378-1127(03)00004-5

[B12] Dai C. C. Yu B. Y. Li X. (2008). Screening of endophytic fungi that promote the growth of *Euphorbia pekinensis*. *Afr. J. Biotechnol.* 7 3505–3510. 10.4314/ajb.v7i19.59361

[B13] Durán P. Thiergart T. Garrido-Oter R. Agler M. Kemen E. Schulze-Lefert P. (2018). Microbial interkingdom interactions in roots promote *Arabidopsis* survival. *Cell* 175 973–983.e14. 10.1016/j.cell.2018.10.020 30388454 PMC6218654

[B14] Esfahani M. Abbasi H. R. A. Rabiei B. Kavousi M. (2008). Improvement of nitrogen management in rice paddy fields using chlorophyll meter (SPAD). *Paddy Water Environ.* 6 181–188. 10.1007/s10333-007-0094-6

[B15] Finkel O. M. Salas-González I. Castrillo G. Conway J. M. Law T. F. Teixeira P. J. (2020). A single bacterial genus maintains root growth in a complex microbiome. *Nature* 587 103–108. 10.1038/s41586-020-2778-7 32999461 PMC10329457

[B16] Gu S. Wei Z. Shao Z. Friman V. P. Cao K. Yang T. (2020). Competition for iron drives phytopathogen control by natural rhizosphere microbiomes. *Nat. Microbiol.* 5 1002–1010. 10.1038/s41564-020-0719-8 32393858 PMC7116525

[B17] Guo Y. Matsuoka Y. Nishizawa T. Ohta H. Narisawa K. (2018). Effects of rhizobium species living with the dark septate endophytic fungus veronaeopsis simplex on organic substrate utilization by the host. *Microb. Environ.* 33 102–106. 10.1264/jsme2.ME17144 29459501 PMC5877336

[B18] Han H. S. Lee K. D. (2006). Effect of co-inoculation with phosphate and potassium solubilizing bacteria on mineral uptake and growth of pepper and cucumber. *Plant Soil Environ.* 52 130–136.

[B19] Harbort C. J. Hashimoto M. Inoue H. Niu Y. Guan R. Rombolà A. D. (2020). Root-secreted coumarins and the microbiota interact to improve iron nutrition in *Arabidopsis*. *Cell Host Microbe* 28 825–837. 10.1016/j.chom.2020.09.006 33027611 PMC7738756

[B20] Harsonowati W. Marian M. Surono M. Narisawa K. (2020). The effectiveness of a dark septate endophytic fungus, *Cladophialophora chaetospira* SK51, to mitigate strawberry Fusarium wilt disease and with growth promotion activities. *Front. Microbiol.* 11:585. 10.3389/fmicb.2020.00585 32351466 PMC7174500

[B21] He C. Wang W. Hou J. (2020). Plant performance of enhancing licorice with dual inoculating dark septate endophytes and *Trichoderma viride* mediated via effects on root development. *BMC Plant Biol.* 20:325. 10.1186/s12870-020-02535-9 32646473 PMC7346674

[B22] Helfrich E. J. N. Vogel C. M. Ueoka R. Schäfer M. Ryffel F. Müller D. B. (2018). Bipartite interactions, antibiotic production and biosynthetic potential of the *Arabidopsis* leaf microbiome. *Nat. Microbiol.* 3 909–919. 10.1038/s41564-018-0200-0 30038309 PMC7115891

[B23] Hermosa R. Viterbo A. Chet I. Monte E. (2012). Plant-beneficial effects of *Trichoderma* and of its genes. *Microbiology* 158 17–25. 10.1099/mic.0.052274-0 21998166

[B24] Hiruma K. Gerlach N. Sacristán S. Nakano R. T. Hacquard S. Kracher B. (2016). Root endophyte *Colletotrichum tofieldiae* confers plant fitness benefits that are phosphate status dependent. *Cell* 165 464–474. 10.1016/j.cell.2016.02.028 26997485 PMC4826447

[B25] Hiruma K. Kobae Y. Toju H. (2018). Beneficial associations between Brassicaceae plants and fungal endophytes under nutrient-limiting conditions: evolutionary origins and host – symbiont molecular mechanisms. *Curr. Opin. Plant Biol.* 44 145–154. 10.1016/j.pbi.2018.04.009 29738938

[B26] Ichihashi Y. Ichihashi Y. Date Y. Date Y. Shino A. Shimizu T. (2020). Multi-omics analysis on an agroecosystem reveals the significant role of organic nitrogen to increase agricultural crop yield. *Proc. Natl. Acad. Sci. U.S.A.* 117 14552–14560. 10.1073/pnas.1917259117 32513689 PMC7321985

[B27] Jansa J. Forczek S. T. Rozmoš M. Püschel D. Bukovská P. Hršelová H. (2019). *Arbuscular mycorrhiza* and soil organic nitrogen: network of players and interactions. *Chem. Biol. Technol. Agric.* 6:10. 10.1186/s40538-019-0147-2

[B28] Kennedy P. G. Peay K. G. Bruns T. D. (2009). Root tip competition among ectomycorrhizal fungi: are priority effects a rule or an exception? *Ecology* 90 2098–2107. 19739372 10.1890/08-1291.1

[B29] Khastini R. O. Ohta H. Narisawa K. (2012). The role of a dark septate endophytic fungus, veronaeopsis simplex Y34, in fusarium disease suppression in Chinese cabbage. *J. Microbiol.* 50 618–624. 10.1007/s12275-012-2105-6 22923110

[B30] Kiers E. T. Duhamel M. Beesetty Y. Mensah J. A. Franken O. Verbruggen E. (2011). Reciprocal rewards stabilize cooperation in the mycorrhizal symbiosis. *Science* 333 880–882. 10.1126/science.1208473 21836016

[B31] Levy A. Salas Gonzalez I. Mittelviefhaus M. Clingenpeel S. Herrera Paredes S. Miao J. (2018). Genomic features of bacterial adaptation to plants. *Nat. Genet.* 50 138–150. 10.1038/s41588-017-0012-9 29255260 PMC5957079

[B32] Lugtenberg B. Kamilova F. (2009). Plant-growth-promoting rhizobacteria. *Ann. Rev. Microbiol.* 63 541–556. 10.1146/annurev.micro.62.081307.162918 19575558

[B33] Lundberg D. S. Lebeis S. L. Paredes S. H. Yourstone S. Gehring J. Malfatti S. (2012). Defining the core *Arabidopsis thaliana* root microbiome. *Nature* 488 86–90. 10.1038/nature11237 22859206 PMC4074413

[B34] Ma L. J. Geiser D. M. Proctor R. H. Rooney A. P. O’Donnell K. Trail F. (2013). Fusarium pathogenomics. *Ann. Rev. Microbiol.* 67 399–416. 10.1146/annurev-micro-092412-155650 24024636

[B35] Manamgoda D. S. Rossman A. Y. Castlebury L. A. Chukeatirote E. Hyde K. D. (2015). A taxonomic and phylogenetic re-appraisal of the genus *Curvularia* (Pleosporaceae): human and plant pathogens. *Phytotaxa* 212 175–198. 10.11646/phytotaxa.212.3.1

[B36] Michielse C. B. Rep M. (2009). Pathogen profile update: *Fusarium oxysporum*. *Mol. Plant Pathol.* 10 311–324. 10.1111/j.1364-3703.2009.00538.x 19400835 PMC6640313

[B37] Nahalkova J. Fatehi J. Olivain C. Alabouvette C. (2008). Tomato root colonization by fluorescent-tagged pathogenic and protective strains of *Fusarium oxysporum* in hydroponic culture differs from root colonization in soil. *FEMS Microbiol. Lett.* 286 152–157. 10.1111/j.1574-6968.2008.01241.x 18657114

[B38] Nara K. (2006). Ectomycorrhizal networks and seedling establishment during early primary succession. *New Phytol.* 169 169–178. 10.1111/j.1469-8137.2005.01545.x 16390428

[B39] Narisawa K. Usuki F. Hashiba T. (2004). Control of verticillium yellows in Chinese cabbage by the dark septate endophytic fungus LtVB3. *Phytopathology* 94 412–418. 10.1094/PHYTO.2004.94.5.412 18943758

[B40] Nelson J. M. Hauser D. A. Hinson R. Shaw A. J. (2018). A novel experimental system using the liverwort *Marchantia polymorpha* and its fungal endophytes reveals diverse and context-dependent effects. *New Phytol.* 218 1217–1232. 10.1111/nph.15012 29411387

[B41] Netto A. T. Campostrini E. de Oliveira J. G. Bressan-Smith R. E. (2005). Photosynthetic pigments, nitrogen, chlorophyll a fluorescence and SPAD-502 readings in coffee leaves. *Sci. Hortic.* 104 199–209. 10.1016/j.scienta.2004.08.013

[B42] Newsham K. K. (2011). A meta-analysis of plant responses to dark septate root endophytes. *New Phytol.* 190 783–793. 10.1111/j.1469-8137.2010.03611.x 21244432

[B43] Nguyen N. H. Song Z. Bates S. T. Branco S. Tedersoo L. Menke J. (2016). FUNGuild: an open annotation tool for parsing fungal community datasets by ecological guild. *Fungal Ecol.* 20 241–248. 10.1016/j.funeco.2015.06.006

[B44] Niki T. Mitsuhara I. Seo S. Ohtsubo N. Ohashi Y. (1998). Antagonistic effect of salicylic acid and jasmonic acid on the expression of pathogenesis-related (PR) protein genes in wounded mature tobacco leaves. *Plant Cell Physiol.* 39 500–507. 10.1093/oxfordjournals.pcp.a029397

[B45] Olivain C. Humbert C. Nahalkova J. Fatehi J. L’Haridon F. Alabouvette C. (2006). Colonization of tomato root by pathogenic and nonpathogenic *Fusarium oxysporum* strains inoculated together and separately into the soil. *Appl. Environ. Microbiol.* 72 1523–1531. 10.1128/AEM.72.2.1523-1531.2006 16461707 PMC1392888

[B46] Paredes H. S. Gao T. Law T. F. Finkel O. M. Mucyn T. Teixeira P. J. P. L. (2018). Design of synthetic bacterial communities for predictable plant phenotypes. *PLoS Biol.* 16:e2003962. 10.1371/journal.pbio.2003962 29462153 PMC5819758

[B47] Peay K. G. Kennedy P. G. Talbot J. M. (2016). Dimensions of biodiversity in the Earth mycobiome. *Nat. Rev. Microbiol.* 14 434–447. 10.1038/nrmicro.2016.59 27296482

[B48] Pieterse C. M. J. Zamioudis C. Berendsen R. L. Weller D. M. van Wees S. C. M. Bakker P. A. H. M. (2014). Induced systemic resistance by beneficial microbes. *Ann. Rev. Phytopathol.* 52 347–375. 10.1146/annurev-phyto-082712-102340 24906124

[B49] Pineda A. Dicke M. Pieterse C. M. J. Pozo M. J. (2013). Beneficial microbes in a changing environment: are they always helping plants to deal with insects? *Funct. Ecol.* 27 574–586. 10.1111/1365-2435.12050

[B50] Priyadharsini P. Muthukumar T. (2017). The root endophytic fungus *Curvularia geniculata* from *Parthenium hysterophorus* roots improves plant growth through phosphate solubilization and phytohormone production. *Fungal Ecol.* 27 69–77. 10.1016/j.funeco.2017.02.007

[B51] R Core Team. (2020). *R: A Language and Environment for Statistical Computing.* Vienna: R Foundation for Statistical Computing.

[B52] Radhakrishnan R. Khan A. L. Kang S. M. Lee I. J. (2015). A comparative study of phosphate solubilization and the host plant growth promotion ability of *Fusarium verticillioides* RK01 and *Humicola* sp. KNU01 under salt stress. *Ann. Microbiol.* 65 585–593. 10.1007/s13213-014-0894-z

[B53] Remy W. Taylor T. N. Hass H. Kerp H. (1994). Four hundred-million-year-old vesicular arbuscular mycorrhizae. *Proc. Nat. Acad. Sci. U.S.A.* 91 11841–11843. 10.1073/pnas.91.25.11841 11607500 PMC45331

[B54] Richardson A. E. Barea J. M. McNeill A. M. Prigent-Combaret C. (2009). Acquisition of phosphorus and nitrogen in the rhizosphere and plant growth promotion by microorganisms. *Plant Soil* 321 305–339. 10.1007/s11104-009-9895-2

[B55] Robert-Seilaniantz A. Grant M. Jones J. D. G. (2011). Hormone crosstalk in plant disease and defense: more than just JASMONATE-SALICYLATE antagonism. *Ann. Rev. Phytopathol.* 49 317–343. 10.1146/annurev-phyto-073009-114447 21663438

[B56] Rudgers J. A. Afkhami M. E. Bell-Dereske L. Chung Y. A. Crawford K. M. Kivlin S. N. (2020). Climate disruption of plant-microbe interactions. *Ann. Rev. Ecol. Evol. Syst.* 51 561–586. 10.1146/annurev-ecolsys-011720-090819

[B57] Saad M. M. Eida A. A. Hirt H. (2020). Tailoring plant-associated microbial inoculants in agriculture: a roadmap for successful application. *J. Exp. Bot.* 71 3878–3901. 10.1093/jxb/eraa111 32157287 PMC7450670

[B58] Schlaeppi K. Bulgarelli D. (2015). The plant microbiome at work. *Mol. Plant Microbe Interact.* 212 212–217. 10.1094/MPMI-10-14-0334-FI 25514681

[B59] Taurian T. Anzuay M. S. Angelini J. G. Tonelli M. L. Ludueña L. Pena D. (2010). Phosphate-solubilizing peanut associated bacteria: screening for plant growth-promoting activities. *Plant Soil* 329 421–431. 10.1007/s11104-009-0168-x

[B60] Taylor T. N. Remy W. Haas H. Kerp H. (1995). Fossil arbuscular mycorrhizae from the early Devonian. *Mycologia* 87 560–573. 10.2307/3760776

[B61] Tedersoo L. May T. W. Smith M. E. (2010). Ectomycorrhizal lifestyle in fungi: global diversity, distribution, and evolution of phylogenetic lineages. *Mycorrhiza* 20 217–263. 10.1007/s00572-009-0274-x 20191371

[B62] Thoms D. Liang Y. Haney C. H. (2021). Maintaining symbiotic homeostasis: how do plants engage with beneficial microorganisms while at the same time restricting pathogens? *Mol. Plant Microbe Interact.* 34 462–469. 10.1094/mpmi-11-20-0318-fi 33534602

[B63] Toju H. Abe M. S. Ishii C. Hori Y. Fujita H. Fukuda S. (2020). Scoring species for synthetic community design: network analyses of functional core microbiomes. *Front. Microbiol.* 11:1361. 10.3389/fmicb.2020.01361 32676061 PMC7333532

[B64] Toju H. Peay K. G. Yamamichi M. Narisawa K. Hiruma K. Naito K. (2018a). Core microbiomes for sustainable agroecosystems. *Nat. Plants* 4 247–257. 10.1038/s41477-018-0139-4 29725101

[B65] Toju H. Tanabe A. S. Sato H. (2018b). Network hubs in root-associated fungal metacommunities. *Microbiome* 6:116. 10.1101/270371 29935536 PMC6015470

[B66] Toju H. Yamamichi M. Guimarães P. R. Jr. Olesen J. M. Mougi A. Yoshida T. (2017). Species-rich networks and eco-evolutionary synthesis at the metacommunity level. *Nat. Ecol. Evol.* 1:54. 10.1038/s41559-016-0024 28812622

[B67] Toju H. Yamamoto S. Tanabe A. S. Hayakawa T. Ishii H. S. (2016). Network modules and hubs in plant-root fungal biomes. *J. R. Soc. Interf.* 13:20151097. 10.1098/rsif.2015.1097 26962029 PMC4843674

[B68] Trivedi P. Leach J. E. Tringe S. G. Sa T. Singh B. K. (2020). Plant–microbiome interactions: from community assembly to plant health. *Nat. Rev. Microbiol.* 18 607–621. 10.1038/s41579-020-0412-1 32788714

[B69] Tsolakidou M. D. Stringlis I. A. Fanega-Sleziak N. Papageorgiou S. Tsalakou A. Pantelides I. S. (2019). Rhizosphere-enriched microbes as a pool to design synthetic communities for reproducible beneficial outputs. *FEMS Microbiol. Ecol.* 95:fiz138. 10.1093/femsec/fiz138 31504462

[B70] Usuki F. Narisawa K. (2007). A mutualistic symbiosis between a dark septate endophytic fungus, *Heteroconium chaetospira*, and a nonmycorrhizal plant, Chinese cabbage. *Mycologia* 99 175–184. 10.3852/mycologia.99.2.175 17682770

[B71] van der Heijden M. G. A. Hartmann M. (2016). Networking in the plant microbiome. *PLoS Biol.* 14:e1002378. 10.1371/journal.pbio.1002378 26871440 PMC4752285

[B72] van der Heijden M. G. A. Klironomos J. N. Ursic M. Moutoglis P. Streitwolf-Engel R. Boller T. (1998). Mycorrhizal fungal diversity determines plant biodiversity, ecosystem variability and productivity. *Nature* 396 69–72. 10.1038/23932

[B73] van Wees S. C. van der Ent S. Pieterse C. M. (2008). Plant immune responses triggered by beneficial microbes. *Curr. Opin. Plant Biol.* 11 443–448. 10.1016/j.pbi.2008.05.005 18585955

[B74] Vinale F. Sivasithamparam K. Ghisalberti E. L. Marra R. Woo S. L. Lorito M. (2008). Trichoderma–plant–pathogen interactions. *Soil Biol. Biochem.* 40 1–10. 10.1016/j.soilbio.2007.07.002

[B75] Vorholt J. A. Vogel C. Carlström C. I. Müller D. B. (2017). Establishing causality: opportunities of dynthetic vommunities for plant microbiome tesearch. *Cell Host Microbe* 22 142–155. 10.1016/j.chom.2017.07.004 28799900

[B76] Wagg C. Schlaeppi K. Banerjee S. Kuramae E. E. van der Heijden M. G. A. (2019). Fungal-bacterial diversity and microbiome complexity predict ecosystem functioning. *Nat. Commun.* 10:117. 10.1038/s41467-019-12798-y 31649246 PMC6813331

[B77] Wang X. Pan Q. Chen F. Yan X. Liao H. (2011). Effects of co-inoculation with arbuscular mycorrhizal fungi and rhizobia on soybean growth as related to root architecture and availability of N and P. *Mycorrhiza* 21 173–181. 10.1007/s00572-010-0319-1 20544230

[B78] Ważny R. Rozpądek P. Jędrzejczyk R. J. Śliwa M. Stojakowska A. Anielska T. (2018). Does co-inoculation of Lactuca serriola with endophytic and arbuscular mycorrhizal fungi improve plant growth in a polluted environment? *Mycorrhiza* 28 235–246. 10.1007/s00572-018-0819-y 29359253 PMC5851704

[B79] Wei Z. Gu Y. Friman V.-P. P. Kowalchuk G. A. Xu Y. Shen Q. (2019). Initial soil microbiome composition and functioning predetermine future plant health. *Sci. Adv.* 5:eaaw0759. 10.1126/sciadv.aaw0759 31579818 PMC6760924

[B80] Werner G. D. A. Kiers E. T. (2015). Order of arrival structures arbuscular mycorrhizal colonization of plants. *New Phytol.* 205 1515–1524. 10.1111/nph.13092 25298030

[B81] Zhu J. Tremblay N. Liang Y. (2012). Comparing SPAD and atLEAF values for chlorophyll assessment in crop species. *Can. J. Soil Sci.* 92 645–648. 10.4141/CJSS2011-100

