# Supplementary material for: Synergistic and Offset Effects of Fungal Species Combinations on Plant Performance
Source: Front Microbiol. 2021 Sep 13;12:713180. doi: 10.3389/fmicb.2021.713180 (PMC8478078; doi:10.3389/fmicb.2021.713180)
Supplement: Supplementary file 1 [file Data_Sheet_1.zip › Data_Sheet/Suppl.Fig_S1.pdf]

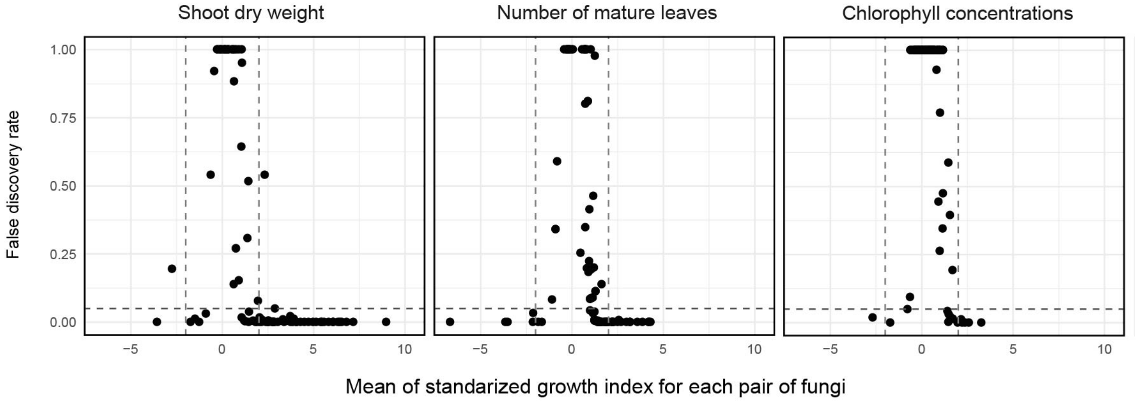

Supplementary Figure S1 | Standardized growth index and its statistical sign. The false discovery rates of *t*-tests between control and target single-/dual-inoculation treatments are plotted against the axis of standardized growth index values averaged across replicate samples.
