# Supplementary figures and images for: Synergistic and Offset Effects of Fungal Species Combinations on Plant Performance
Source: Front Microbiol. 2021 Sep 13;12:713180. doi: 10.3389/fmicb.2021.713180 (PMC8478078; doi:10.3389/fmicb.2021.713180)

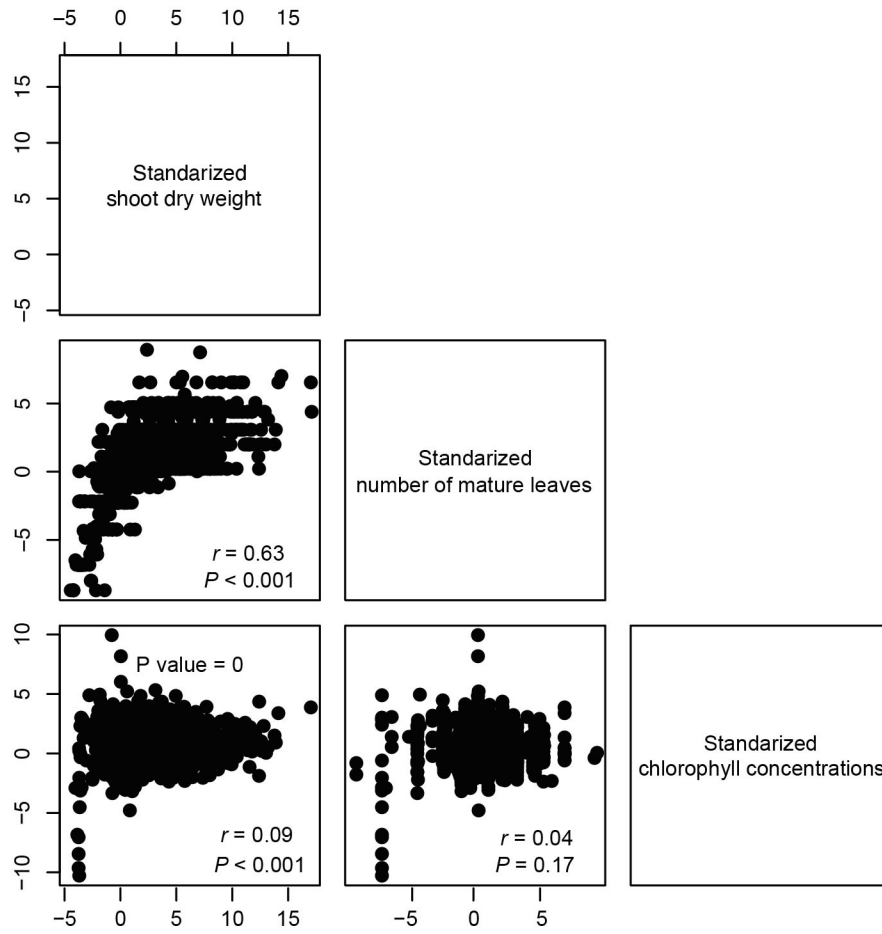

**Supplementary Figure S3 | Relationships among plant performance traits.**

Supplement: Supplementary file 1 [file Data_Sheet_1.zip › Data_Sheet/Suppl.Fig_S3.pdf]
